# Emergency psychiatry services in pandemia: Is it different than before?

**DOI:** 10.1192/j.eurpsy.2021.1873

**Published:** 2021-08-13

**Authors:** İ.G. Yılmaz Karaman, L.D. Tarlacık

**Affiliations:** Psychiatry Department, Eskişehir Osmangazi University, Faculty of Medicine, Eskişehir, Turkey

**Keywords:** Suicide, emergency psychiatry, Pandemia, COVID-19

## Abstract

**Introduction:**

After World Health Organization declared that COVID-19 disease became a pandemic; like most, people in Turkey were affected by the emotionally challenging atmosphere. Previous outbreaks negatively effected mental health, increased suicide attempts and completed suicides.

**Objectives:**

Our study aimed to investigate psychiatry consultations from emergency service in a university hospital, to determine differencies in pandemia.

**Methods:**

We conducted a monocenter retrospective study by examining the patients who applied to emergency servise and consulted to psychiatry department in three periods: between 11 March- 11 July, in 2018, 2019, and 2020. Patient’s sociodemographic and clinical variables were assessed.

**Results:**

**Figure:**
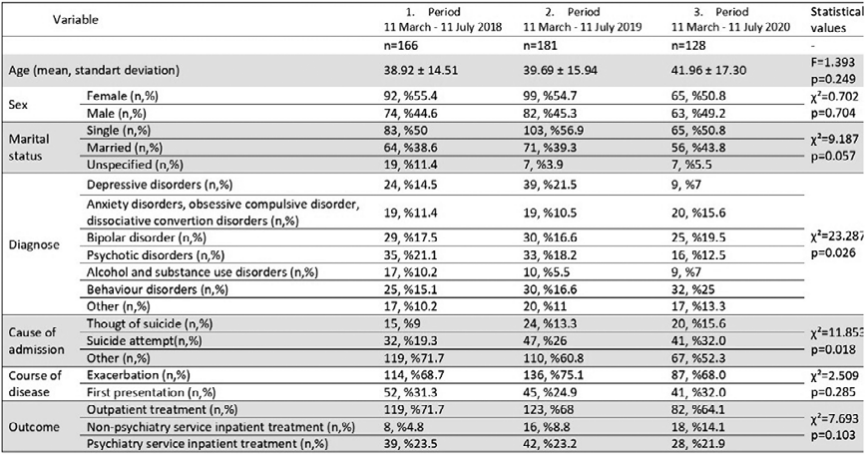

**Figure:**
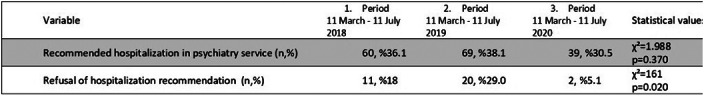

There were no difference in distributions of applicants’ following variables between periods; age, sex, marital status, experiencing a first attack or an exacerbation, or outcome treatment. Among applicants with suicide attempts, there were no difference between periods in terms of the presence of recurrent suicide attempt (χ² = 0.297 p = 0.862). While emergency admissions with behavioral disorders increased, admissions with depressive symptoms decreased. Admissions with suicide attempts were statistically significantly higher in 2020 (Table 1). Recommendation of psychiatric inpatient treatment did not change between periods, while refusal of hospitalization recommendation decreased (Table 2).

**Conclusions:**

In our sample, emergency psychiatry admissions with behaviour disorders and suicide attempts increased in pandemic period.

**Disclosure:**

No significant relationships.

